# Robust Crop and Weed Segmentation under Uncontrolled Outdoor Illumination

**DOI:** 10.3390/s110606270

**Published:** 2011-06-10

**Authors:** Hong Y. Jeon, Lei F. Tian, Heping Zhu

**Affiliations:** 1 United State Department of Agriculture—Agricultural Research Service, Application Technology Research Unit, 1680 Madison Ave, Wooster, OH 44691, USA; E-Mail: heping.zhu@ars.usda.gov; 2 Agricultural and Biological Engineering Department, University of Illinois, 1304 W. Pennsylvania Ave., Urbana, IL 61801, USA; E-Mail: lei-tian@illinois.edu

**Keywords:** field crop, machine vision, outdoor illumination, weed identification

## Abstract

An image processing algorithm for detecting individual weeds was developed and evaluated. Weed detection processes included were normalized excessive green conversion, statistical threshold value estimation, adaptive image segmentation, median filter, morphological feature calculation and Artificial Neural Network (ANN). The developed algorithm was validated for its ability to identify and detect weeds and crop plants under uncontrolled outdoor illuminations. A machine vision implementing field robot captured field images under outdoor illuminations and the image processing algorithm automatically processed them without manual adjustment. The errors of the algorithm, when processing 666 field images, ranged from 2.1 to 2.9%. The ANN correctly detected 72.6% of crop plants from the identified plants, and considered the rest as weeds. However, the ANN identification rates for crop plants were improved up to 95.1% by addressing the error sources in the algorithm. The developed weed detection and image processing algorithm provides a novel method to identify plants against soil background under the uncontrolled outdoor illuminations, and to differentiate weeds from crop plants. Thus, the proposed new machine vision and processing algorithm may be useful for outdoor applications including plant specific direct applications (PSDA).

## 1. Introduction

The limited accuracy of the delivery of agrochemicals to targeted areas is one of the inherent issues of broadcast application technique, thus, broadcast application has great potential of over- and under-applications [1]. However, plant specific direct applications (PSDA) which directly apply a predetermined agrochemical amount to an individual weed or weed infested area is known to reduce herbicide use, and increase application efficiency for weed control. PSDA potentials have been addressed in some studies [2–7] because the PSDA is an much more efficient way of using herbicides compared to broadcast application.

One of the challenges for the PSDA is to detect plants and identify weeds in field, and it is a demanding issue because it is an inherently complex process due to the wide species varibility, non-uniform structures, and plant shape changes as they grow. Very limited techniques are available to detect weeds against crop plants and soil in the field, including remote sensing [8–10], spectral reflectance [11–13] and machine vision [5,14–19]. The spatial and temporal resolutions of remote sensing limit the technique for real-time PSDA. The spectral reflectance technique relies on weed infestation levels in the sensing area, making it insufficient for detecting an individual weed or low infestation levels due to the low spatial resolution. Machine vision shows the greatest potential to overcome the limitations of other systems due to its superior spatial resolution [20]. Thus, the machine vision is a feasible sensing technique for the PSDAs.

One of the common issues with the machine vision technique in the field applications is to overcome the often unpredictable and non-uniform (shadows in the field of view) outdoor illumination because unpredictable and non-uniform illumination in the field of view directly affect the captured image quality. As a result, unless the machine vision system has a dynamic segmentation algorithm, it is common to produce undesired segmentation results. Therefore, plant segmentation algorithms for machine vision have been investigated to robustly detect plants under outdoor conditions. For example, an environmentally adaptive segmentation algorithm (EASA) with an automated look-up table (LUT) generator was used to identify tomato cotyledons from field images under variable illumination conditions [21]. However, the algorithm only correctly identified 45 to 67% of tomato cotyledons in the images.

The use of two algorithms to segment sunny and shadow parts of an image has been attempted [22]. The authors used a luminance threshold value to identify either sunny or shadow pixels, and identified pixels were processed by either sunny or shadow pixel segmentation algorithms. The accuracy of their algorithms was not mentioned, however, they reported 78.3% and 90.3% classification rates for sugar beet and weeds, respectively.

A filtered-background subtraction to eliminate shadow effects in detecting a seed row was used to detect it with Hough transformation and monochrome images [23]. However, this method uses image binning to reduce computational load and may limit the spatial accuracy for weed detection. In addition, using monochrome images may be vulnerable to detect plants under shadow conditions.

Research in using a combination of RGB (Red-Green-Blue) channels to identify weeds has been reported. For example, the Excessive Green (EG) method has been used to segment plant images against soil [5,14,17]. Although the results of the plant segmentation were successful, their segmentation process required manual adjustment which is a major hurdle for autonomous field applications. Because inconsistent field illumination influences the color information of field images, research in segmenting plants against non-plant areas via transforming RGB channels to HSI (Hue-Saturation-Intensity) spaces was carried out. A common strategy of this research in detecting weeds was to build a look-up table [4,6] for identifying weeds in transformed images. The methods resulted in marginal success detecting weeds and crop plants (68.8% and 73.1% for crop plants and weeds, respectively).

Although image segmentation algorithms have limitations for autonomous PSDA systems, further efforts have been made to identify weeds against crop plants using machine vision. For example, an attempt at using an ANN (Artificial Neural Network) to identify broadleaf and grass weeds was made with forty plant images [17]. Gabor wavelets were used to extract plant features from the images. Twenty randomly selected images were used to train the ANN, and the other images were used to validate the ANN, which was able to classify either the broadleaf or grass weeds from the validation image set.

The ANN technique has also been applied to classify corn plant images captured under natural illumination [24]. The images were captured in variable natural lighting conditions, and they were cropped and modified to minimize computational efforts. Each image contained only a single plant that was rotated by 90, 180 and 270 degrees to build a robust ANN by introducing various oriented plant images. The identification rate for corn plants was reported to be 100% while an identification range of 62–92% was reported for weeds.

A machine vision system with two weed identification methods, ANN and discriminant analysis, was developed and evaluated in a radish farm [25]. The authors used 150 field images, 50 radish and 100 weed images, and the identification rates of the discriminant analysis were 92 and 98% for radish plant and weed, respectively. ANN showed 100% identification rate for radish plants and weed. However, weed detection methods from early studies required manual processing to improve image quality and plant detail. Manual processing is a critical limitation for an autonomous PSDA system in machine vision applications. Thus, the objective of this study was to develop a vegetation detection algorithm with an adaptive image segmentation for autonomous PSDA systems, and for identification of early stage crop plants (late VE (emergence) to V1 (first leaf)) against weeds and soil from field images under uncontrolled outdoor illumination conditions.

## 2. Materials and Methods

### 2.1. Machine Vision

**Figure 1.**
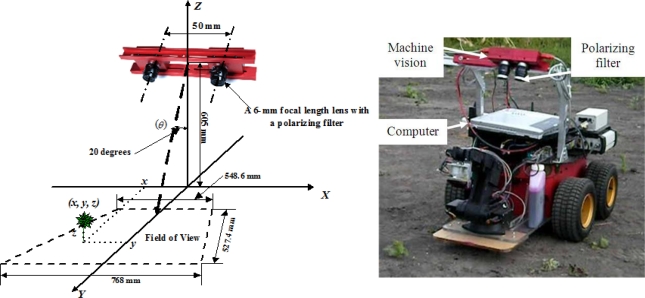
Schematic description of the machine vision installation (**left**) and actual installation of the machine vision on a field robot (**right**). XY plane is the ground.

A left RGB (Red-Green-Blue) sterovision camera (STH-MDCS2-VAR-C, Videre Design, Menlo Park, CA, USA) was used as a machine vision system. The imaging sensor of the machine vision unit was a half-inch color complementary metal-oxide-semiconductor (CMOS; MT9M001, pixel size: 5.2 × 5.2 μm, Micron, Boise, ID, USA). An IEEE 1394 (FireWire) interface was used to communicate between the camera and the computer that controlled the machine vision system. The image resolution of 320 × 240 pixels was selected to minimize image processing efforts. The camera was equipped with a C-mount lens (LM6NCL, F = 1.4, Kowa Co., Nagoya, Japan) with a focal length of 6-mm (horizontal viewing angle: 58.1° and vertical viewing angle: 45.2°). A liner polarizing filter (VF-25CPKS, Sony Co. LTD, Tokyo, Japan) was attached in front of the lens to prevent specular reflectance from intense outdoor illumination. The installation of the machine vision may be seen in Figure 1.

The height of the machine vision was approximately 605 mm from the ground, and the machine vision was angled at 20 degrees with respect to the ground surface normal. It was mounted on upside-down L shape frame, and the frame was attached to a skid steering robot (PC3-AT, ActivMedia Inc., Amberst, NH, USA). The field of view of the camera was a trapezoid and its area was approximately 548.6 (768.0) mm × 572.4 mm (W_lower_ W_upper_) × H, resolution: 1.7 (2.4) mm/pixel × 2.4 mm/pixel).

### 2.2. Image Capturing and Processing

Each camera in the machine vision system was calibrated using its built-in functions [26], and the calibration parameters were loaded to the machine vision unit. A manually controlled field robot carried the system, and the robot has a curved-fiberglass solar panel roof to supply the power to the robot. Thus, the machine vision viewing area had multiple illuminations, sunny and shady, because the solar panel created shadow from direct sunlight in the robot’s front area (0.45 m^2^). Lens aperture and focus were manually adjusted in the field through visual examination, and the gain and exposure of the machine vision were set by visual examination as well. A C++ program was written to capture field images and manually control the field robot. Field images of early stage corn plants were collected by the machine vision system on May 14th and 18th, 2007 at a cornfield in the Agricultural Engineering Research Farm at the University of Illinois. A total of 1,278 images were captured on the morning of May 14th, and 998 images on the afternoon of May 18th, 2007, thus, the two sets of field images had different illumination conditions and growth stages. The field image was processed in Matlab (The MathWorks, Natick, MA, USA) to detect weeds with following methods: image normalization, adaptive image segmentation, noise filtering and individual plant identification.

Each field image was converted to normalized red (R)-, green (G)- and blue (B)-channel images:

(1) $$R=\frac{r}{r+g+b},G=\frac{g}{r+g+b},B=\frac{b}{r+g+b}$$

where, r, g, and b are a pixel value of red, green and blue channel of RGB image.

The normalized RGB channels were converted to the normalized excessive green (NEG) images [27,28], and the constants for the conversion were modified to emphasizing green channel:

(2) NEG=2.8⋅G−R−B

**Figure 2.**
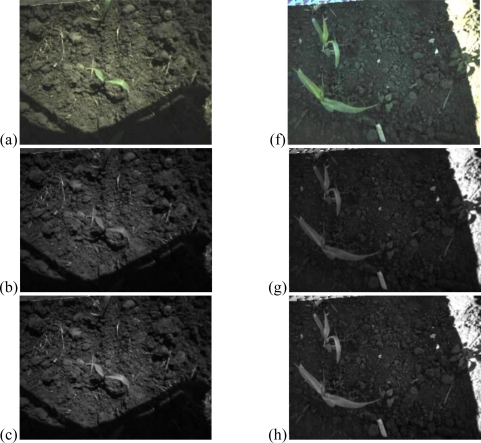

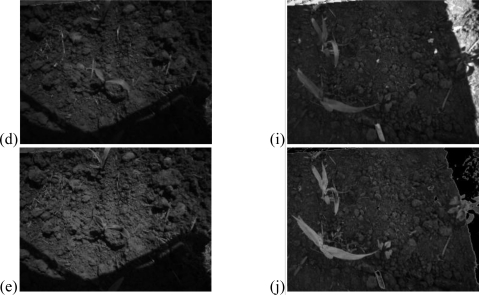
An example of original, red-, green-, blue-channel and normalized excessive green images. (**a**) Field image captured in the morning. (**b**) Red channel of the morning image. (**c**) Green channel of the morning image. (**d**) Blue channel of the morning image. (**e**) Normalized excessive-green image of the morning image. (**f**) Field image captured in the afternoon (**g**) Red channel of the afternoon image. (**h**) Green channel of the afternoon image. (**i**) Blue channel of the afternoon image. (**j**) Normalized excessive-green image of the afternoon image.

NEG pixel values were converted to integer values via multiplying by 100 to NEG for image histogram computation. Conversion example is in Figure 2 (the image brightness is increased by 10% for better visual representation).

The threshold value of each NEG image to segment the plant against soil was automatically determined by dividing the pixel distribution of the image to two groups by a pixel value ranging from 1 to 255. The pixel value that minimizes the variance sum of two groups was selected as the threshold value of each image [29]. To minimize random noise and plant segmentation errors, following empirical criteria were applied to autonomously adjust the threshold values:

1. If the segmented pixels are less than 20% (15,360 pixels) of the total image pixels (76,800 pixels), upper image segmentation limit (R_L_), the algorithm moves on to the next image process step. Otherwise, the threshold value is adjusted by an increment (or decrement).
2. If the segmented pixels are more than 0.5% (384 pixels) of the total image pixels, lower image segmentation limit (RL), algorithm moves on to the next step of the image process. Otherwise, the threshold value is adjusted by an increment (or decrement).
3. The increment for adjusting the threshold value is determined:
  (3) $$\text{Increment}\;(\text{decrement})=\text{Round}\left(\frac{R_{C}-R_{L}}{R_{L}}\times 20\right)$$
  where R_C_: Rate (%) of segmented pixels with respect to total pixels of the image with current threshold and R_L_: Upper (When the initial rate of image segmentation is greater than 20%) or lower (When the initial rate of image segmentation is lower than 0.5%) limits of image segmentation in a percentage with respect to the total image pixels.
4. The lower limit will change to 5% (3,840 pixels) when the initial rates of the segmented pixels are less than 0.5%.

**Figure 3.**
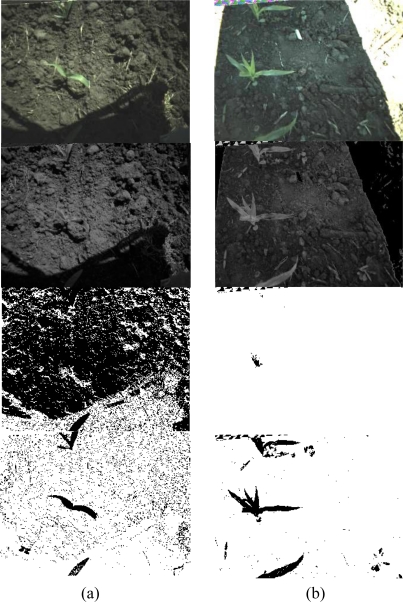
Changes of image segmentation results by adjusting the threshold values: two conditions of initial image segmentation results are presented. (**a**) Changes of image segmentation results by adjusting threshold values when the initial segmented pixel rate was higher than the upper limit (Images shown are original RGB (top), NEG, 1st and 2nd (bottom) threshold adjustment images). (**b**) Changes of image segmentation results by adjusting threshold value when the initial segmented pixel rate was smaller than the lower limit (Images shown are original RGB (top), NEG, 4th and 9th (bottom) threshold adjustment images).

The maximum adjustments of the threshold value were limited to 30 to prevent a continuous loop of the adjustments, and the increment for the threshold value adjustment was adaptively changed by the distance between the current segmented pixel rates and the image segmentation limit. Thus, overshooting adjustment near the limits was prevented so that abrupt changes on image segmentation from adjusting the threshold values were avoided. In addition, since the current algorithm was designed with typical corn field spacing (row spacing of 81 cm) and relatively light plant density (less than 20% of imaging area), updating segmentation limits may be required for higher plant density fields. Image segmentation examples are seen in Figure 3: one was higher than the upper limit and the other was lesser than the lower limit (Figure 3; the field image brightness is increased by 10% for better visual representation).

A 3-by-3 median filter was applied to segmented images to eliminate random noise in the image. The image processing algorithm was verified with four image groups: randomly selected stationary (68) and non-stationary images (231) captured on the 14th of May 2007, and also randomly selected stationary (178) and non-stationary images (189) captured on the 18th of May 2007. Stationary images were collected while the robot was at a stationary position in the field, and non-stationary images were collected while the robot was carrying the machine vision through the field. They were processed automatically via the developed algorithm without manual adjustments, and the errors of image processing during the automated process were identified under variable outdoor illuminations. The results of image processing were evaluated by examining processed gray scale images with the original RGB images. The processing error in segmenting vegetation from the soil background was reported, and the definition of the error is total number of incorrectly identified plants over total plants in each image group.

### 2.3. Weed and Crop Plant Identification

The ANN was selected to identify weeds from crop plants from field images since ANN was a determined to be a successful method to identify crop plants from field images [24,25]. Since weeds have relatively higher variability in their morphological features, identifying crop plants was used as a strategy of identifying weeds from field images. To train and verify the ANN, the machine vision and a digital camera (SD110, Canon, Tokyo, Japan) were used to capture corn and weed images, including cocklebur (*Xanthium Strumarium*), common lambs quarters (*Chenopodium album*), morning glory (*Ipomoea*) and velvetleaf (*Abutilon theophrasti*), in the field, greenhouse and laboratory. Images for training and verifying ANN were preprocessed to extract accurate morphological features: weeds and corn images were copied from the images, and they were pasted on a uniform background.

**Figure 4.**
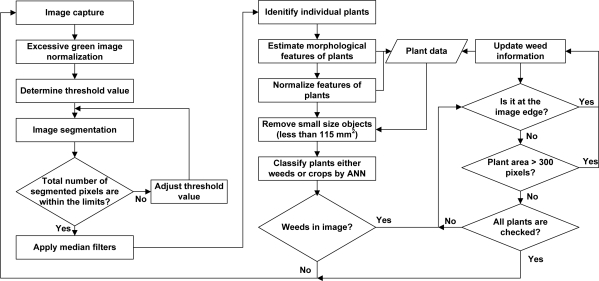
Flowchart for the image processing algorithm.

A total of 240 non-occluded images (109 corn and 131 weed images) were collected and prepared to train and verify the ANN. Four morphological features of plants in pixel unit were measured: (1) the plant perimeter (PRI) defines the perimeter of a plant, (2) the area defines the inner area of a plant; (3) the width, and (4) the height define the longest and shortest distance within a plant, respectively. Four features were then converted to five normalized features: height/width, height/PRI, PRI/area, width/area, and height/area. Thus, the influences of plant image sizes were minimized to plants’ morphological features. Normalized features of plants were divided into two groups for ANN training (54 corn and 65 weed feature data) and verification (55 corn and 66 weed feature data). The two hidden layer ANN with 5 and 1 neuron structures was used to identify weeds against crop plants because the ANN showed the highest identification rate. Matlab was used to train and validate the network. Image processing algorithm with the validated ANN was written in Matlab, and the algorithm automatically processed two sets of field images to simulate autonomous field application conditions. Its identification accuracy and potential improvement were discussed. The flowchart of the overall image processing for the machine vision system is shown in Figure 4.

## 3. Results and Discussion

### 3.1. Image Processing

**Figure 5.**
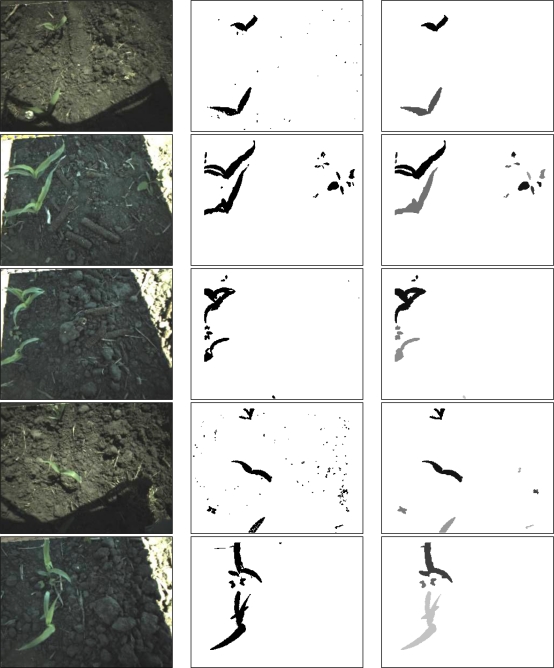
Example results of image processing: raw field image (**left column**), plant segmentation (**middle column**), and individual plant identification (different plants have different gray scale intensity, **right column**).

Raw field images, segmented image and individual plant detection results, are shown in Figure 5 (the field image brightness increased by 10% for better visual representation). The results of the image processing had a processing error of 2.9% in stationary field images for individual plant identification (Table 1, right column in Figure 5; the different intensities in the image refers to different plants). The processing error was 1.7 to 2.6% for the non-stationary field images even though field images had multiple illuminations.

The plant detection results were stable over the illumination condition changes of the imaging area (middle column in Figure 5). The algorithm detected plants against a soil background under different illuminations (inside and outside of the solar panel shade areas) although the plants were under sunny and shade areas or the shade area (1st and 4th row in Figure 5). However, the plant detection using the algorithm exposed its limitations in the saturated image area simply because field images did not contain any notable variations in R, G and B pixel intensities. The other aspect that should be noted was when two plants were occluded, the algorithm could not separate them but considered them as a single plant (3rd row in Figure 5).

### 3.2. Weed and Crop Plant Identification

The identification rate of 72.6% was reported from image set 1: ANN correctly identified 345 out of 475 corn plants (Table 2). Sources of the incorrect identification were investigated in the image, and the main source was identified to be the incomplete corn plant images at the edge of the field images (16.2%). To improve the identification accuracy, two criteria were applied to improve the identification results of the ANN by minimizing the error in the corn plant identification. The first criterion was to ignore the identification results of the ANN for the plant at the edges of the image. Thus, the plant identification results with incomplete morphological features were excluded from the identification. The other was to set a maximum weed size in pixel: the maximum size of a weed was limited to 300 pixels which was determined in a preliminary study.

**Figure 6.**
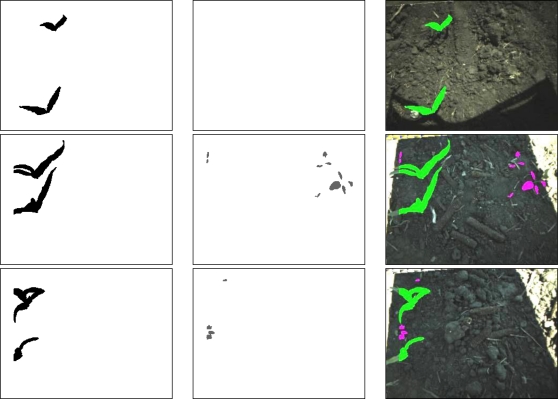

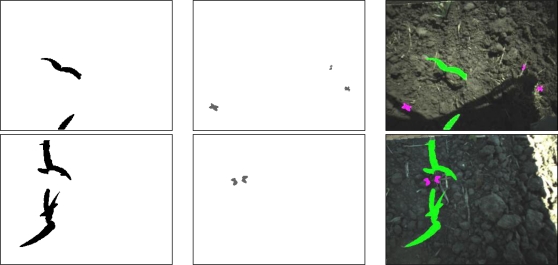
Example result images of weed identification: image with corn plants (**left column**), image with weeds (**middle row**) and field image with identified weeds (purple) and corns (green) (**right column**).

The identification rates of the corn plants increased to 92.5% for image set 1 (231 field images) and 95.1% for image set 2 (189 field images) by implementing the two criteria (Table 2). The ANN correctly identified 444 out of the 480 corn plants in field image set 1 and 388 out of the 408 corn plants in field image set 2. Examples of corn and weed identification from the field images are shown in Figure 6 (Image brightness increases by 10% for better visual representation).

The developed ANN showed better performance in identifying corn plants and weeds in the field. For example, the developed ANN identified more than 90% of corn plants in field images in the automated process. However, identification rates using a Bayesian classifier [4] were 73.1% and 68.8% for tomato plants and weeds, respectively, in field images although the classifier used 10 plant features. Minimum distance function (MDF), the minimum distance from the centroid to edge, was used, and its accuracy in detecting crop plants was 78.7% of crop plants in field images [5].

Higher identification rates have been achieved with ANN [25]. However, their ANN was trained with more plant features (eight features) to identify weeds, and plant morphological features were manually extracted in their research. On the other hand, the ANN developed in this study identified crop plants from field images with automatically extracted normalized morphological features. Thus, the results and evaluation methods showed herein that developed weed detection method has great potential in autonomously identifying the crop plants under uncontrolled field illuminations by processing a series of field images with multiple illuminations.

## 4. Conclusions

A machine vision system with weed detection and an adaptive image segmentation algorithm was developed and tested. The algorithm contained adaptive image segmentation to robustly identify plants under uncontrolled outdoor illuminations. Thus, the weed detection algorithm presented here was relatively robust against outdoor illumination changes, and it may be relatively stable for outdoor application use. A series of field images was processed without manual adjustments to detect plants: images were processed via normalized excessive green process, computing threshold value, adaptive image segmentation, and median filter. The normalized morphological features of plants were autonomously computed from the processed images, and the features were supplied to the trained ANN to identify weeds and crop plants in the images. Results of the image processing show that the developed segmentation algorithm had the processing errors of 2.9% for stationary images and of 2.1% for non-stationary images with illumination changes. The trained ANN identified 72.6% of the corn plants in the field images. A major source of the inaccuracy, incomplete corn plants at the image edges, was addressed in the identification process, and improved identification rates of 92.5% and 95.1% were achieved for two image sets.

## Figures and Tables

**Table 1.** Image processing error for individual plant identification.

	**Stationary field images**		**Non-Stationary field images**	
	**Image group 1**	**Image group 2**	**Image group 1**	**Image group 2**
A number of processed images	68	178	231	189

A rate of processing error (%)	2.9	0	1.7	2.6

**Table 2.** Evaluation of the ANN’s performance on identification of corn plants.

**Image set description**	**Corn plant identification rate (%)**			
	**Without filters**			**With filters**
	**Correct**	**Errors**		
		**Edges**	**Rest**	
Image set 1 (Morning of May 14, 2007)	72.6	16.2	11.2	92.5

Image set 2 (Afternoon of May 18, 2007)	-	-	-	95.1
